# The effect of autologous platelet-rich plasma intrauterine perfusion on pregnancy outcomes of frozen-thawed embryo transfer in patients with chronic endometritis

**DOI:** 10.3389/frph.2025.1644445

**Published:** 2025-08-11

**Authors:** Lili Chen, Lan Liu, Huanhuan Guo, Zhenhua Wang

**Affiliations:** ^1^ Reproductive Medicine Center, Affiliated Hospital of Putian University, Putian, Fujian, China; ^2^ Department of Cardiology, The Second Affiliated Hospital of Fujian Medical University, Quanzhou, Fujian, China

**Keywords:** chronic endometritis, autologous platelet-rich plasma, frozen-thawed embryo transfer, live birth rate per transfer cycle, clinical pregnancy rate per transfer cycle

## Abstract

**Objective:**

To analyze the effect of autologous platelet-rich plasma (PRP) intrauterine perfusion on the clinical outcomes of frozen-thawed embryo transfer in patients with chronic endometritis.

**Methods:**

A retrospective continuous cohort of 219 patients diagnosed with chronic endometritis at the Reproductive Medicine Center of Affiliated Hospital of Putian University between January 2020 and December 2022, undergoing their first frozen-thawed embryo transfer cycle, was included. All patients received standardized oral doxycycline treatment after diagnosis. Based on whether they received PRP intrauterine perfusion prior to embryo transfer, patients were divided into an observation group (PRP group, *n*=103) and a control group (non-PRP group, *n* = 116). Outcome measures included live birth rate per transfer cycle, clinical pregnancy rate per transfer cycle, and early miscarriage rate per clinical pregnancy.

**Results:**

All patients underwent endometrial preparation using a hormone replacement therapy (HRT) protocol. Endometrial thickness on the day before transformation was significantly higher in the PRP group compared to the control group (10.58 ± 1.78 mm vs. 9.79 ± 1.58 mm, *P* = 0.001). The PRP group exhibited significantly higher clinical pregnancy rate per transfer cycle and live birth rate per transfer cycle than the control group (58.25% vs. 40.52%, *P* = 0.038; 52.43% vs. 34.48%, *P* = 0.007). The difference in early miscarriage rate per clinical pregnancy between the PRP group and the control group was not statistically significant (8.33% vs. 14.89%, *P* = 0.86). The live birth rate per transfer cycle in the single-PRP subgroup was significantly lower than in the multiple-PRP subgroup (44.62% vs. 65.79%, *P* = 0.038). There were no statistically significant differences in clinical pregnancy rate per transfer cycle or early miscarriage rate per clinical pregnancy between the single-PRP and multiple-PRP subgroups (52.31% vs. 68.42%, *P* = 0.110; 11.76% vs. 3.85%, *P* = 0.377).

**Conclusion:**

For patients diagnosed with chronic endometritis undergoing their first frozen-thawed blastocyst transfer after standardized antibiotic treatment, adjunctive PRP intrauterine perfusion therapy improves pregnancy outcomes.

## 1 Introduction

Chronic endometritis (CE) is a persistent, non-specific chronic inflammatory disease of the endometrium, characterized by abnormal plasma cell infiltration in the endometrial stroma. Recent studies indicate that CE is closely associated with conditions such as intrauterine adhesions, endometrial polyps, and endometriosis. Furthermore, research has demonstrated that CE is also linked to an increased risk of adverse pregnancy outcomes, including infertility, recurrent implantation failure (RIF), and recurrent pregnancy loss (1).

Epidemiological data report CE prevalence rates of 2.8%–56.8% among infertile patients (2), with oral antibiotics as the standard treatment. Studies report a clinical cure rate as high as 89.0% with antibiotic therapy, alongside improvements in pregnancy outcomes (3). However, controversy exists regarding whether antibiotic treatment improves pregnancy outcomes in CE, as some studies report no significant increase in pregnancy or live birth rates for CE patients following oral antibiotics (1). Therefore, beyond endometrial immune-inflammatory imbalance, CE may impact embryo implantation through multiple pathways, including impaired endometrial decidualization and compromised endometrial receptivity.

PRP is prepared from autologous blood, with a platelet concentration 3–5 times higher than baseline plasma levels. It is rich in growth factors, cytokines, and other bioactive molecules (4). Intrauterine perfusion of PRP can promote endometrial repair, reduce the production of pro-inflammatory factors, and improve endometrial receptivity, garnering significant clinical interest. Previous research has primarily focused on analyzing the benefits of PRP therapy in improving pregnancy outcomes during embryo transfer cycles for patients with thin endometrium or RIF. However, only a limited number of studies have investigated the effect of PRP on embryo transfer outcomes specifically in patients with endometritis. This study aims to investigate the impact of PRP therapy on the outcomes of frozen-thawed blastocyst transfer in patients experiencing their first implantation failure, who have received antibiotic treatment and are undergoing their first artificial cycle frozen-thawed embryo transfer (FET).

## 2 Materials and methods

### 2.1 Study subjects and grouping

**Inclusion Criteria:** (1) Met the diagnostic criteria for CE: Positive CD138 immunostaining on endometrial histopathology, followed by standardized treatment with doxycycline (100 mg twice daily for 14 days) starting the day after diagnosis. (2) Age ≤ 37 years. (3) Availability of at least one high-quality D5/D6 blastocyst (graded as stage 4 or above with both inner cell mass and trophectoderm scoring B or higher according to the Gardner scoring system).

**Exclusion Criteria:** (1) Chromosomal abnormality in either partner. (2) Uterine malformation, intrauterine adhesions, submucosal fibroids, endometrial polyps, or endometrial hyperplasia. (3) Untreated hydrosalpinx or recurrent hydrosalpinx after previous treatment. (4) Moderate to severe endometriosis. (5) Immunological diseases such as antiphospholipid syndrome. (6) Thyroid dysfunction. (7) History of recurrent pregnancy loss or RIF.

Clinical data from 219 patients diagnosed with CE at the Reproductive Medicine Center of the Affiliated Hospital of Putian University between January 2020 and December 2022, undergoing their first FET cycle, were retrospectively and consecutively included. Based on whether they received PRP intrauterine perfusion, patients were divided into an observation group (PRP group, *n* = 103) and a control group (*n* = 116), defined as follows: (1) Observation group: PRP intrauterine perfusion group, subdivided into single-session PRP intrauterine perfusion group and multiple-session (≥2) PRP intrauterine perfusion group based on the number of PRP treatments. (2) Control group: Non-PRP intrauterine perfusion group. This study was approved by the Ethics Committee of the Affiliated Hospital of Putian University (Approval No. 2025174), and conducted in accordance with the local legislation and institutional requirements. All patients provided their written informed consent to participate in this study.

### 2.2 FET clinical protocol

**Control Group:** Hormone replacement therapy (HRT) protocol was used. Patients started oral estradiol/estradiol dydrogesterone tablets (Femoston red tablets, 2 mg/tablet, Abbott Healthcare Products B.V., Netherlands) at 4–6 mg/day beginning on day 2–3 of the menstrual cycle or withdrawal bleeding. The estradiol dose was adjusted based on endometrial thickness, aiming for a thickness ≥8 mm before endometrial transformation. Endometrial transformation was achieved using progesterone vaginal sustained-release gel (Crinone, 90 mg/tube, Merck Serono GmbH, Germany) at 1 tube/day. All patients underwent single high-quality frozen-thawed blastocyst transfer on day 6 after transformation initiation. Luteal phase support consisted of oral estradiol dydrogesterone tablets (Femoston yellow tablets, 2 mg/tablet, Abbott Healthcare Products B.V., Netherlands) at 6 mg/day combined with progesterone vaginal gel (1 tube/day) continued until 14 days post-transfer.

**Observation Group:** The HRT protocol was identical to the control group. PRP intrauterine perfusion was performed starting 2 days after the cessation of menstruation and up to the day before endometrial transformation. Luteal phase support was identical to the control group.

### 2.3 PRP preparation method and intrauterine perfusion

**PRP Preparation:** PRP was prepared from the patient's autologous peripheral blood using a two-step centrifugation method. 20 ml of venous blood was drawn into a collection tube containing sodium citrate anticoagulant. After centrifugation at 300 × g for 10 min, the upper platelet-rich supernatant was collected. This supernatant was centrifuged again at 800 × g for 10 min. The supernatant was discarded, leaving approximately 1 ml of precipitated platelet plasma (PRP). The PRP was immediately mixed and activated with 20 µl of thrombin (Thrombin 2 ml/500 IU; Hunan Yige Pharmaceutical Co., Ltd.) prior to perfusion.

**PRP Intrauterine Perfusion:** The patient was placed in the lithotomy position. After routine disinfection and draping, the processed PRP solution was drawn into a 1 ml syringe connected to an intrauterine insemination (IUI) catheter. The catheter was passed through the cervix, and the PRP solution was slowly injected into the uterine cavity. The catheter was left in place for 30 s before being gently removed. The patient remained in a supine position with hips elevated for 15 min post-procedure.

### 2.4 Outcome measures

**Pregnancy Indicators:** Clinical Pregnancy: Defined as the visualization of an intra- or extra-uterine gestational sac by ultrasound 5 weeks after embryo transfer. Early Miscarriage: Defined as fetal loss before 12 weeks of gestation. Live Birth: Defined as the delivery of a live infant after 28 weeks of gestation. Clinical Pregnancy Rate per Transfer Cycle = (Number of clinical pregnancies/Number of transfer cycles) × 100%. Early Miscarriage Rate per clinical pregnancy = (Number of early miscarriages/Number of clinical pregnancies) × 100%. Live Birth Rate per Transfer Cycle = (Number of live births/Number of transfer cycles) × 100%.

**Ultrasound Indicators:** Endometrial thickness was measured using a GE Voluson E6 color Doppler ultrasound system (General Electric Company, USA) on the day before transformation and the day before embryo transfer.

### 2.5 Statistical analysis

Statistical analysis was performed using SPSS software (version 26.0). Continuous variables were tested for normality using the Shapiro–Wilk test. Normally distributed data were expressed as mean ± standard deviation (x¯ ± s) and compared between groups using the independent samples *t*-test. Non-normally distributed data were expressed as median (interquartile range, IQR; P25, P75) and compared using the Mann–Whitney *U* test. Categorical data were expressed as frequency [percentage, *n* (%)] and analyzed using the Chi-square test or Fisher's exact test. A *P*-value < 0.05 was considered statistically significant.

## 3 Results

### 3.1 Comparison of baseline characteristics between groups

I. The total of 219 patients consecutively diagnosed with CE and undergoing their first frozen-thawed blastocyst transfer cycle were included. Among them, 103 patients were in the PRP group and 116 in the control group. There were no statistically significant differences between the two groups in terms of age, body mass index (BMI), duration of infertility, type of infertility, causes of infertility, basal follicle-stimulating hormone (FSH) level, or basal estradiol (E2) level(all *P* > 0.05). Details are presented in Table 1.

**Table 1:** Comparison of baseline characteristics of patients undergoing frozen-thawed blastocyst transfer between PRP group and control group.

Variable	PRP Group (*n* = 103)	Control Group (*n* = 116)	Statistic (*t*/*z*/*χ*² Value)	*P*
Transfer cycles (*n*)	103	116	-	-
Female age (years)	30.18 ± 3.49	30.66 ± 3.17	−1.05	0.297
BMI (kg/m²)	21.44 ± 2.80	21.28 ± 2.63	0.43	0.668
Duration of infertility (years)	4 (2, 5)	3 (2, 5)	−0.76	0.446
Type of infertility (%)			0.19	0.661
Primary	49.51 (51/103)	46.55 (54/116)		
Secondary	50.49 (52/103)	53.44 (62/116)		
Basal FSH (IU/L)	6.87 ± 1.62	7.06 ± 2.36	−0.72	0.474
Basal E2 (pmol/L)	33.80 (26.90, 42.40)	35.55 (27.10, 45.38)	−0.99	0.323
Cause of infertility (%)			2.40	0.493
Male factor	30.10 (31/103)	35.34 (41/116)		
Female factor	52.43 (54/103)	43.10 (50/116)		
Combined factor	11.65 (12/103)	12.07 (14/116)		
Unexplained	5.83 (6/103)	9.48 (11/116)		

### 3.2 Comparison of pregnancy outcomes between groups

The results showed that compared to the control group, the PRP group had significantly higher clinical pregnancy rate per transfer cycle (*P* = 0.038) and live birth rate per transfer cycle (*P* = 0.007). The PRP group showed a lower early miscarriage rate per clinical pregnant (8.33%) compared to controls (14.89%), though this difference was not statistically significant (*P* = 0.286). Details are presented in Table 2.

**Table 2:** Comparison of pregnancy outcomes between PRP group and control group.

Variable	PRP Group (*n* = 103)	Control Group (*n* = 116)	χ²	*P*-value
Clinical pregnancy rate per transfer cycle	58.25 (60/103)	40.52 (47/116)	4.31	0.038*
Early miscarriage rate per clinical pregnant	8.33 (5/60)	14.89 (7/47)	1.14	0.286
Live birth rate per transfer cycle	52.43 (54/103)	34.48 (40/116)	7.17	0.007**

Statistically significant: *p < 0.05, **p < 0.01.

### 3.3 Comparison of endometrial thickness between groups

The results showed that endometrial thickness on the day before progesterone administration was significantly greater in the PRP group compared to the control group (*P* = 0.001). However, there was no statistically significant difference in endometrial thickness on the day of transfer between the two groups (*P* > 0.05). The change in endometrial thickness from the day before progesterone administration to the day of transfer differed between groups. A decrease in thickness indicated endometrial compaction. The incidence of endometrial compaction was significantly higher in the PRP group (33 cases) than in the control group (21 cases) (*P* = 0.017). Details are presented in Table 3.

**Table 3:** Comparison of endometrial thickness between PRP group and control group.

Variable	PRP Group (*n* = 103)	Control Group (*n* = 116)	Statistic (*t*/χ² Value)	*P*
Endometrial thickness day before *P* (mm)	10.58 ± 1.78	9.79 ± 1.58	3.46	0.001**
Endometrial thickness on transfer day (mm)	10.32 ± 1.85	10.29 ± 1.97	0.11	0.915
Endometrial compaction (%)	32.04 (33/103)	18.10 (21/116)	5.70	0.017*

Statistically significant: *p < 0.05, **p < 0.01.

### 3.4 Comparison of pregnancy outcomes based on PRP perfusion frequency

Clinical pregnancy rate per transfer cycle, early miscarriage rate per clinical pregnant, and live birth rate per transfer cycle according to the number of PRP perfusions are shown in Table 4. The results showed that live birth rate per transfer cycle increased significantly with an increasing number of PRP perfusions (*P* = 0.038). While not reaching statistical significance, PRP treatment showed favorable directional trends: increased clinical pregnancy rates per transfer cycle and decreased miscarriage rates per clinical pregnancy.

**Table 4:** Comparison of pregnancy outcomes based on number of PRP perfusions.

Variable	Single PRP (*n* = 65)	Multiple PRP (*n* = 38)	Statistic (χ^2^/Fisher)	*P*
Clinical pregnancy rate per transfer cycle	52.31 (34/65)	68.42 (26/38)	2.56	0.110
Early miscarriage rate per clinical pregnant	11.76 (4/34)	3.85 (1/26)	-	0.377^a^
Live birth rate per transfer cycle	44.62 (29/65)	65.79 (25/38)	4.31	0.038*

Statistically significant: *p < 0.05.

^a^ Fisher's exact test was used for comparison (no χ^2^ value reported).

## 4 Discussion

This study aimed to investigate the effect of intrauterine perfusion of PRP on pregnancy outcomes in patients with CE undergoing FET cycles using a HRT protocol. The results demonstrated that PRP administration significantly improved clinical pregnancy rates, live birth rates, and endometrial thickness on the day before progesterone administration compared to controls—findings consistent with prior research on PRP applications in infertility management (5, 6). Notably, our results revealed a novel dose-response relationship: increasing numbers of PRP perfusions correlated with progressively higher clinical pregnancy and live birth rates within our specific patient cohort.

CE represents a persistent inflammatory state of the endometrium that adversely impacts embryo implantation, contributing to infertility and poor pregnancy outcomes. Patients with CE exhibit abnormal plasma cell infiltration within the endometrial stroma. However, conventional hematoxylin and eosin (H&E) staining for histopathological diagnosis relies on morphological identification of plasma cells, which introduces subjective interpretation bias. This method frequently leads to confusion between plasma cells, endometrial stromal fibroblasts, and monocytes, while both round and spindle-shaped plasma cells may be overlooked—factors collectively compromising CE diagnosis. CD138 (syndecan-1), as a specific plasma cell marker, has gained widespread clinical adoption in recent years due to the high sensitivity and technical simplicity of its immunohistochemical (IHC) staining. Current evidence indicates CD138 IHC is a reliable diagnostic method that significantly improves CE detection accuracy (7). While CE is primarily caused by intrauterine microbial infections requiring confirmation through endometrial culture or molecular pathogen detection, empirical antibiotic therapy remains standard clinical practice due to limitations in current microbiological testing methodologies. Doxycycline, with its broad antimicrobial spectrum, is recommended as first-line treatment based on clinical studies demonstrating efficacy against CE (8). Consequently, this study utilized CD138+ IHC staining to diagnose CE through plasma cell detection, followed by standardized doxycycline antibiotic treatment initiated the day after diagnosis.

Since Chang et al. (9) first demonstrated in 2015 that PRP intrauterine perfusion improved clinical pregnancy outcomes in patients with thin endometrium, subsequent studies have consistently shown its benefit in enhancing pregnancy outcomes in FET cycles. However, the majority of these studies focused on patients with thin endometrium, thin endometrium combined with RIF, or RIF alone (10–13), with only a limited number addressing FET patients specifically with concurrent CE. Sfakianoudis et al. (14) reported on a woman with CE and RIF who experienced another failure after the first antibiotic treatment. Persistent CE was confirmed after the second antibiotic course. Following PRP treatment, the patient achieved clinical cure of CE and subsequently conceived twins with a successful delivery after embryo transfer. A study by Li et al. (6), which included RIF patients with CE, found that combined antibiotic and PRP intrauterine perfusion helped improve clinical pregnancy and live birth rates in FET cycles, but the early miscarriage rate was high (12.70%). Our study observed similar positive effects on pregnancy and live birth rates, but differed in finding a lower early miscarriage rate (8.33%) with a decreasing trend. This discrepancy may be attributed to differences in the study populations and the quality of embryos transferred.

Endometrial thickness reflects endometrial receptivity. PRP intrauterine perfusion can enhance endometrial thickness and receptivity, providing a more favorable environment for embryo implantation. Multiple studies have demonstrated that PRP perfusion increases endometrial thickness in patients with thin endometrium (10, 15). Similarly, our results showed that the PRP group had significantly greater endometrial thickness on the day before progesterone administration compared to the control group (10.58 ± 1.78 mm vs. 9.79 ± 1.58 mm, *P* = 0.001). Recent research suggests that endometrial compaction (a decrease in thickness after progesterone administration) may be a better indicator of receptivity than thickness alone (16). Therefore, we further investigated whether PRP perfusion influenced endometrial compaction. Our results indicate a significantly higher incidence of endometrial compaction in the PRP group compared to the control group (32.04% vs. 18.10%, *P* = 0.017). Continued endometrial growth after progesterone administration may be associated with progesterone insufficiency or resistance. Consequently, endometrial compaction signifies superior endometrial receptivity.

CE is characterized by abnormal plasma cell infiltration in the endometrial stroma, alterations in immune cell subpopulations and cytokines leading to an altered immune microenvironment, impaired endometrial receptivity, and disrupted signaling at the maternal-fetal interface, ultimately contributing to implantation failure. PRP intrauterine perfusion may improve pregnancy outcomes in CE patients through several mechanisms: (1) Immune Microenvironment Modulation: CE alters the endometrial immune milieu. PRP perfusion can shift the peripheral blood Th1/Th2 cell balance towards Th2 dominance (17, 18), potentially enhancing clinical pregnancy rates. (2) Anti-inflammatory Effects: CE induces a pro-inflammatory state, characterized by increased pro-inflammatory cytokines and decreased anti-inflammatory factors (19). PRP, rich in growth factors, plays a key regulatory role, reducing the expression of pro-inflammatory markers such as interleukin-1β (IL-1β), tumor necrosis factor (TNF), and matrix metalloproteinases (MMPs) (20). Platelets within PRP interact with immune cells, reducing leukocyte infiltration (21). Furthermore, platelets modulate immune factors like transforming growth factor-β (TGF-β), platelet factor 4 (PF4), and neutrophil-activating peptide-2 (NAP-2), thereby attenuating inflammation (22). (3) Enhanced Endometrial Receptivity and Vascularization: CE causes receptivity defects, manifesting as aberrant estrogen receptor expression in glands, displacement of the implantation window, impaired decidualization, and uterine hypoxia/abnormal blood supply (23). PRP perfusion, via growth factors like platelet-derived growth factor (PDGF), vascular endothelial growth factor (VEGF), and insulin-like growth factor (IGF), promotes endometrial proliferation, vascular remodeling, and improved endometrial blood flow (24). Studies also indicate that PRP treatment upregulates the expression of genes associated with endometrial receptivity (25).

In a randomized controlled trial (RCT) by Nazari et al. (26) involving FET patients with thin endometrium, endometrial thickness significantly increased after the second PRP perfusion (7.213 ± 0.188 mm) compared to after the first (5.993 ± 0.701 mm). Given this dose-dependent effect on thickness, we investigated whether multiple PRP perfusions further improved pregnancy outcomes. Our results show that multiple PRP perfusions yielded a significantly higher live birth rate per transfer cycle compared to a single perfusion (65.79% vs. 44.62%). The underlying mechanisms warrant further investigation.

To minimize confounding factors, this study included only patients aged ≤37 years undergoing transfer of high-quality D5/D6 blastocysts, thereby mitigating the influence of age and embryo quality on outcomes. Several limitations should be acknowledged: (1) The retrospective design limits the strength of our conclusions, although efforts were made to exclude potential confounding biases. (2) The study was conducted at a single center and utilized a non-randomized, retrospective design, which limits the generalizability of our findings. Future multi-center, prospective RCTs are necessary for confirmation. (3) Although our diagnostic approach relied on CD138 immunohistochemistry and standard histopathological evaluation, routine screening for specific infectious agents (e.g., Mycobacterium tuberculosis) was not performed. This limitation may have resulted in undetected cases, underscoring the need for future studies to incorporate broader diagnostic panels. Furthermore, while CD138 serves as a valuable plasma cell marker, its variable sensitivity and specificity suggests supplemental testing—such as pathogen-specific PCR—could enhance diagnostic accuracy in select clinical contexts. (4) The use of a conventional PRP preparation method resulted in a smaller sample size for the multiple-PRP subgroup. While trends towards differences in clinical pregnancy and miscarriage rates were observed between single and multiple PRP groups, they did not reach statistical significance, potentially due to enlarged standard errors subject to the much smaller multiple-PRP sample size.

This study represents a valuable initial exploration. Future research should utilize apheresis systems for PRP preparation. Apheresis offers advantages in efficiency, precision, and supports red blood cell return, enhancing the feasibility of performing multiple PRP intrauterine perfusions clinically. This approach would permit more rigorous examination of the relationship between PRP perfusion frequency and pregnancy outcomes.

## 5 Conclusions

For patients with chronic endometritis, adjunctive PRP intrauterine perfusion following antibiotic treatment significantly improves clinical pregnancy and live birth rates in frozen-thawed embryo transfer cycles. Multiple PRP treatments may further optimize outcomes. We recommend future exploration of apheresis-based PRP preparation to enable efficient collection of larger volumes, allowing for multiple PRP perfusions prior to FET in CE patients, rather than relying solely on antibiotics. Prospective studies are needed to further refine PRP perfusion protocols and preparation techniques.

## Data Availability

The raw data supporting the conclusions of this article will be made available by the authors, without undue reservation.

## References

[1] Johnston-MacAnanny EB Hartnett J Engmann LL Nulsen JC Sanders MM Benadiva CA. Chronic endometritis is a frequent finding in women with recurrent implantation failure after *in vitro* fertilization. Fertil Steril. (2010) 93(2):437–41. 10.1016/j.fertnstert.2008.12.131 19217098

[2] Kimura F Takebayashi A Ishida M Nakamura A Kitazawa J Morimune A Review: chronic endometritis and its effect on reproduction. J Obstet Gynaecol Res. (2019) 45(5):951–60. 10.1111/jog.13937 30843321

[3] Buzzaccarini G Vitagliano A Andrisani A Santarsiero CM Cicinelli R Nardelli C Chronic endometritis and altered embryo implantation: a unified pathophysiological theory from a literature systematic review. J Assist Reprod Genet. (2020) 37(12):2897–911. 10.1007/s10815-020-01955-8 33025403 PMC7714873

[4] Aydin O Karaca G Pehlivanli F Altunkaya C Uzun H Özden H Platelet-rich plasma may offer a new hope in suppressed wound healing when compared to mesenchymal stem cells. J Clin Med. (2018) 7(6):143. 10.3390/jcm7060143 29890683 PMC6025374

[5] Boychuk AV Kotsabyn NV Yakymchuk JB Nikitina IM. Pregravid preparation of women with chronic endometritis in IVF cycles. Wiad Lek. (2024) 77(1):25–8. 10.36740/WLek202401103 38431803

[6] Li J Li X Ding J Zhao J Chen J Guan F Analysis of pregnancy outcomes in patients with recurrent implantation failure complicated with chronic endometritis. Front Cell Dev Biol. (2023) 11:1088586. 10.3389/fcell.2023.1088586 36861040 PMC9969095

[7] Ughade PA Shrivastava D. Unveiling the role of endometrial CD-138: a comprehensive review on its significance in infertility and early pregnancy. Cureus. (2024) 16(2):e54782. 10.7759/cureus.54782 38529432 PMC10961243

[8] Kitaya K Matsubayashi H Takaya Y Nishiyama R Yamaguchi K Takeuchi T Live birth rate following oral antibiotic treatment for chronic endometritis in infertile women with repeated implantation failure. Am J Reprod Immunol. (2017) 78(5):e12719. 10.1111/aji.12719 28608596

[9] Chang Y Li J Chen Y Wei L Yang X Shi Y Autologous platelet-rich plasma promotes endometrial growth and improves pregnancy outcome during *in vitro* fertilization. Int J Clin Exp Med. (2015) 8(1):1286–90. 25785127 PMC4358582

[10] Shin SY Chung N Shin JE Kim JH Park C Kwon H Angiogenic factor-driven improvement of refractory thin endometrium with autologous platelet-rich plasma intrauterine infusion in frozen embryo transfer cycles. Front Endocrinol (Lausanne). (2024) 15:1431453. 10.3389/fendo.2024.1431453 39290323 PMC11405219

[11] Xu Y Hao C Fang J Liu X Xue P Miao R. Intrauterine perfusion of autologous platelet-rich plasma before frozen-thawed embryo transfer improves the clinical pregnancy rate of women with recurrent implantation failure. Front Med (Lausanne). (2022) 9:850002. 10.3389/fmed.2022.850002 35425782 PMC9001903

[12] Enatsu Y Enatsu N Kishi K Otsuki J Iwasaki T Okamoto E Clinical outcome of intrauterine infusion of platelet-rich plasma in patients with recurrent implantation failure. Reprod Med Biol. (2022) 21(1):e12417. 10.1002/rmb2.12417 34938145 PMC8656680

[13] Russell SJ Kwok YSS Nguyen TT-TN Librach C. Autologous platelet-rich plasma improves the endometrial thickness and live birth rate in patients with recurrent implantation failure and thin endometrium. J Assist Reprod Genet. (2022) 39(6):1305–12. 10.1007/s10815-022-02505-0 35508692 PMC9068225

[14] Sfakianoudis K Simopoulou M Nitsos N Lazaros L Rapani A Pantou A Successful implantation and live birth following autologous platelet-rich plasma treatment for a patient with recurrent implantation failure and chronic endometritis. In Vivo. (2019) 33(2):515–21. 10.21873/invivo.11504 30804135 PMC6506282

[15] Yu T-N Lee T-H Lee M-S Chen Y-C Chen C-I Cheng E-H Intrauterine infusion and hysteroscopic injection of autologous platelet-rich plasma for patients with a persistent thin endometrium: a prospective case-control study. J Clin Med. (2024) 13(10):2838. 10.3390/jcm13102838 38792379 PMC11122516

[16] Haas J Smith R Zilberberg E Nayot D Meriano J Barzilay E Endometrial compaction (decreased thickness) in response to progesterone results in optimal pregnancy outcome in frozen-thawed embryo transfers. Fertil Steril. (2019) 112(3):503–509.e1. 10.1016/j.fertnstert.2019.05.001 31248618

[17] Kitazawa J Kimura F Nakamura A Morimune A Hanada T Amano T Alteration in endometrial helper T-cell subgroups in chronic endometritis. Am J Reprod Immunol. (2021) 85(3):e13372. 10.1111/aji.13372 33155317

[18] Chang Y Peng J Zhu Y Sun P Mai H Guo Q How platelet-rich plasma (PRP) intra-uterine injection improve endometrial receptivity of intrauterine adhesions in women: a time-series-based self-controlled study. J Reprod Immunol. (2023) 156:103796. 10.1016/j.jri.2023.103796 36696783

[19] Oshina K Kuroda K Nakabayashi K Tomikawa J Kitade M Sugiyama R Gene expression signatures associated with chronic endometritis revealed by RNA sequencing. Front Med (Lausanne). (2023) 10:1185284. 10.3389/fmed.2023.1185284 37547609 PMC10400718

[20] Cole BJ Karas V Hussey K Merkow DB Pilz K Fortier LA. Hyaluronic acid versus platelet-rich plasma: a prospective, double-blind randomized controlled trial comparing clinical outcomes and effects on intra-articular biology for the treatment of knee osteoarthritis. Am J Sports Med. (2017) 45(2):339–46. 10.1177/0363546516665809. Erratum in: Am J Sports Med. 2017;45(5):NP10. doi: 10.1177/0363546517700110. 28146403

[21] Khatab S van Buul GM Kops N Bastiaansen-Jenniskens YM Bos PK Verhaar JA Intra-articular injections of platelet-rich plasma releasate reduce pain and synovial inflammation in a mouse model of osteoarthritis. Am J Sports Med. (2018) 46(4):977–86. 10.1177/0363546517750635 29373806

[22] Nurden AT. The biology of the platelet with special reference to inflammation, wound healing and immunity. Front Biosci (Landmark Ed). (2018) 23(4):726–51. 10.2741/4613 28930569

[23] Liu Z Liu X Li F Sun Y Yu L Zhang W Overexpression of hypoxia-inducible factor 1α and excessive vascularization in the peri-implantation endometrium of infertile women with chronic endometritis. Front Endocrinol (Lausanne). (2022) 13:1001437. 10.3389/fendo.2022.1001437 36531509 PMC9751377

[24] Puente Gonzalo E Alonso Pacheco L Vega Jiménez A Vitale SG Raffone A Laganà AS. Intrauterine infusion of platelet-rich plasma for severe Asherman syndrome: a cutting-edge approach. Updates Surg. (2021) 73(6):2355–62. 10.1007/s13304-020-00828-0 32514742

[25] Huniadi A Zaha IA Naghi P Stefan L Sachelarie L Bodog A Autologous platelet-rich plasma (PRP) efficacy on endometrial thickness and infertility: a single-centre experience from Romania. Medicina (Kaunas). (2023) 59(9):1532. 10.3390/medicina59091532 37763650 PMC10533168

[26] Nazari L Salehpour S Hoseini S Zadehmodarres S Azargashb E. Effects of autologous platelet-rich plasma on endometrial expansion in patients undergoing frozen-thawed embryo transfer: a double-blind RCT. Int J Reprod Biomed. (2019) 17(6):445–8. 10.18502/ijrm.v17i6.4816 PMC6719514 31508569

